# The life cycle of meridional heat flux peaks

**DOI:** 10.1002/qj.4249

**Published:** 2022-02-23

**Authors:** Andrea Marcheggiani, Maarten H. P. Ambaum, Gabriele Messori

**Affiliations:** ^1^ Department of Meteorology University of Reading Reading UK; ^2^ Department of Earth Sciences and Centre of Natural Hazards and Disaster Science (CNDS) Uppsala University Uppsala Sweden; ^3^ Department of Meteorology and Bolin Centre for Climate Research Stockholm University Stockholm Sweden

**Keywords:** atmosphere, meridional heat flux, midlatitudes, Rossby waves, synoptic, weather regimes

## Abstract

Covariance between meridional wind and air temperature in the lower troposphere quantifies the poleward flux of dry static energy in the atmosphere; in the midlatitudes, this is primarily realised by baroclinic weather systems. It is shown that strong covariance between temperature and meridional wind results from both enhanced correlation and enhanced variance, and that the two evolve according to a distinct temporal structure akin to a life‐cycle. Starting from a state of low correlation and variance, there is first a gradual build‐up to modal growth at constant, high correlation, followed by a rapid decay at relatively low correlation values. This life‐cycle evolution is observed most markedly over oceanic regions, and cannot be explained on purely statistical grounds. We find that local peaks of meridional heat flux are not exclusively linked to the action of individual weather systems and can affect the atmospheric circulation on larger length‐scales through wave propagation along waveguides.

## Graphical abstract

In this paper, we explore the dynamical mechanisms that lead to meridional heat flux peaks and we contextualise them within the life cycle of meridional‐wind—temperature covariance. We find that an increase in correlation and subsequent amplification of variances are associated on average with baroclinic system development, Rossby waves propagation and, more generally, the North Atlantic storm track life cycle.

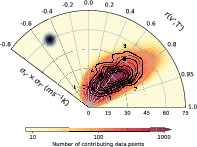

## 1 INTRODUCTION

Meridional heat fluxes^1^in the midlatitudes can be viewed as the climate system's response to the thermal imbalance originating from the differential radiative heating between the Equator and the poles Poleward of roughly $30^{\circ }$ latitude, the atmosphere accounts for the bulk of this flux. In the seminal work by Lorenz (1955) on the energetics of the atmospheric circulation, the meridional flux (or transport) of heat is associated with a conversion term of zonal available potential energy to eddy available potential energy, which can be thought of as the main energy reservoir for midlatitude weather systems to feed on. More recent estimates of the sign and magnitude of this conversion term (Peixoto and Oort, 1992) confirm this view, meaning that meridional heat fluxes play a central role in shaping storm‐track dynamics.

Early work by Swanson and Pierrehumbert (1997) first highlighted the importance played by sporadic transient events of extreme heat flux activity in setting the climatological‐mean heat transport. Specifically, Messori and Czaja (2013) later showed that, every season, only a few days of peak meridional heat transport associated both with baroclinic systems and planetary‐scale motions (Messori and Czaja, 2014) can account for more than half of the net seasonal transport. Messori and Czaja (2015) further explored the mechanisms behind the occurrence of these local extreme events, which, in storm‐track regions, are found to correspond primarily to synoptic structures akin to warm conveyor belts.

The intermittency observed in meridional heat transport extremes was linked to the energy available to weather systems to develop and evolve by Novak *et al*. (2017), who demonstrated the existence of a predator–prey relationship between meridional heat flux and baroclinicity (taken as a local measure of available potential energy) with the help of a nonlinear oscillator model for storm‐track variability (Ambaum and Novak, 2014).

More recently, Marcheggiani and Ambaum (2020) explored the use of spatial covariance between surface heat‐flux and temperature as a descriptor of local air–sea thermal interactions, which in the Lorenz energetics scheme can be associated with diabatic generation or reduction of transient available potential energy. It was found that these air–sea heat fluxes also feature bursts of activity comparable to those in meridional heat fluxes and, in particular, it was observed that strong covariance resulted from a concomitant increase of both correlation and variances in heat flux and temperature. Analogously, we can interpret covariance between meridional wind speed (*v*) and air temperature (*T*) as a measure of local meridional heat transport, which in the Northern Hemisphere is climatologically positive, but can locally attain large negative values depending on season and spatio‐temporal scale of the relevant disturbances (Lembo *et al*., 2019).

Midlatitude storm tracks are typically identified by maxima in either eddy kinetic or available potential energy, which can be measured, respectively, by time variance in meridional wind, $\bar{v^{\prime 2}}$ (with the bar indicating a time average), and temperature, $\bar{T^{\prime 2}}$. Their covariation in time, $\bar{v^{\prime }T^{\prime }}$, represents the conversion of background potential energy to transient available potential energy and, as such, is also associated with storm track intensity. These statistics provide a coherent large‐scale picture, yet present differences in the exact location and extent of the storm track, suggesting that the latter's structure and life cycle cannot be fully explained by variance alone.

Schemm and Rivière (2019) highlighted the importance of the efficiency of transient eddies in extracting energy from the background baroclinicity. Their definition of eddy efficiency is based upon the dot product between the vector fields of eddy heat flux and background baroclinicity. In particular, an efficiency equal to 1 corresponds to a flow configuration whereby baroclinic conversion of eddy available potential energy into eddy kinetic energy is maximised. Schemm and Rivière (2019) surmised that the anomalous poleward tilt with height of eddies entering the North Pacific storm track through its northern seeding branch, which makes them less efficient, is partly responsible for the observed midwinter suppression of storm‐track activity (Nakamura, 1992). In fact, a lower level of correlation is associated with a non‐optimal spatial configuration of synoptic eddies whose damping effect on the temperature spatial variance is not as strong as in the case of higher‐correlated systems, which can instead have a larger impact on the local available potential energy.

In this paper, we explore the idea that the correlation between *v* and *T* and their variances can be used to probe the dynamics of the meridional heat transport, and carry information about the evolution of midlatitude weather systems and storm tracks. The overarching aim of our study is to add detail to the meaning of variance and, by focusing on the evolution of correlation, to isolate the contribution to eddy kinetic energy that is associated with conversion from eddy available potential energy (and measured by covariance between meridional wind and temperature).

In this framework, we observe a concurrent increase of correlation and variances in the build‐up to strong $v^{\prime }$–$T^{\prime }$ covariance (our notation for the spatial covariance between $v^{\prime }$ and $T^{\prime }$; analogously, for *v*–*T* time covariance), corresponding to sporadic heat flux events, and then dissect the distinct roles of variance and correlation in contributing to these events. The analysis indicates the importance of modal growth in the initial phase and uncorrelated decay in the final phase of an event, according to a well‐defined life‐cycle evolution.

The paper is structured as follows. Section 2 illustrates the relationship between time correlation and variances over the North Atlantic ocean. Section 3 introduces the particular space–time framework in which we study the evolution of the spatial covariance between *v* and *T*. In Section 4 we then describe the life cycle of $v^{\prime }$–$T^{\prime }$ covariance through the study of the phase space of its components and explore the link to the evolution of weather systems. Finally, in Section 5 we provide a summary of our results and discuss their implications.

## 2 PROPERTIES OF TIME CORRELATION AND VARIANCE

The climatological average of meridional heat transport was found to be shaped primarily by sporadic extreme events of limited longitudinal and temporal extent (Messori and Czaja, 2013; 2014; Messori *et al*., 2017). These events can be associated with a stronger spatial correlation between *v* and moist static energy time anomalies, which typically characterise baroclinic, or ‘weather’, synoptic systems.

Efficient meridional transport of the dry static energy component relies on a strong correlation between *v* and *T*. A positive correlation between *v* and *T* is usually expected to occur in the Northern Hemisphere, as northerly and southerly winds contribute to the advection of cold and warm air respectively. Therefore, the covariance between *v* and *T* can be interpreted as a measure of the strength of meridional heat transport as, the larger and more positively correlated *v* and *T* anomalies become, the larger is the poleward heat transport.

Covariance between *v* and *T* (either in time or in space) is defined as the product of correlation r between *v* and *T* and their standard deviations σ,

(1) $$\text{cov}(v,T)=r(v,T)\sigma_{v}\sigma_{T}.$$

A related statistic which we often refer to in this study is the variance of *v* and *T*, which is the square of standard deviation.

Our study focuses on the boreal winter season (December, January and February) and is based upon data from the European Centre for Medium‐Range Weather Forecast (ECMWF) Re‐Analysis Interim dataset (ERA‐Interim; Dee *et al*., 2011), spanning winters from 1979 to 2019 with a time resolution of 6 hr and interpolated onto a 1.5${}^{\circ }\times 1.5^{\circ }$ longitude–latitude spatial grid. Meridional wind speed *v* and air temperature *T* at the 850 hPa level are considered.

In the computation of time covariance, correlation and standard deviations, time anomalies are defined as departures from a running mean with a time window of 10 days, as opposed to simply removing the climatological mean. The time covariance between *v* and *T*, for example, is computed as

(2) $$\text{cov}(v,T)=\frac{1}{N}\underset{i}{\sum }(v_{i}-{\hat{v}}_{i})(T_{i}-{\hat{T}}_{i})=\frac{1}{N}\underset{i}{\sum }v_{i}^{\prime }T_{i}^{\prime },$$

where *N* is the total number of time steps *i* and ${\hat{v}}_{i},{\hat{T}}_{i}$ indicate the 10‐day running means of *v* and *T* evaluated at times *i*. This allows us to filter out any lower‐frequency variability not associated with synoptic systems (Athanasiadis and Ambaum, 2009) without excessively manipulating the data, given the simplicity of the time filter implemented. Throughout this study, no further time filtering is applied to the original data.

**FIGURE 1:**
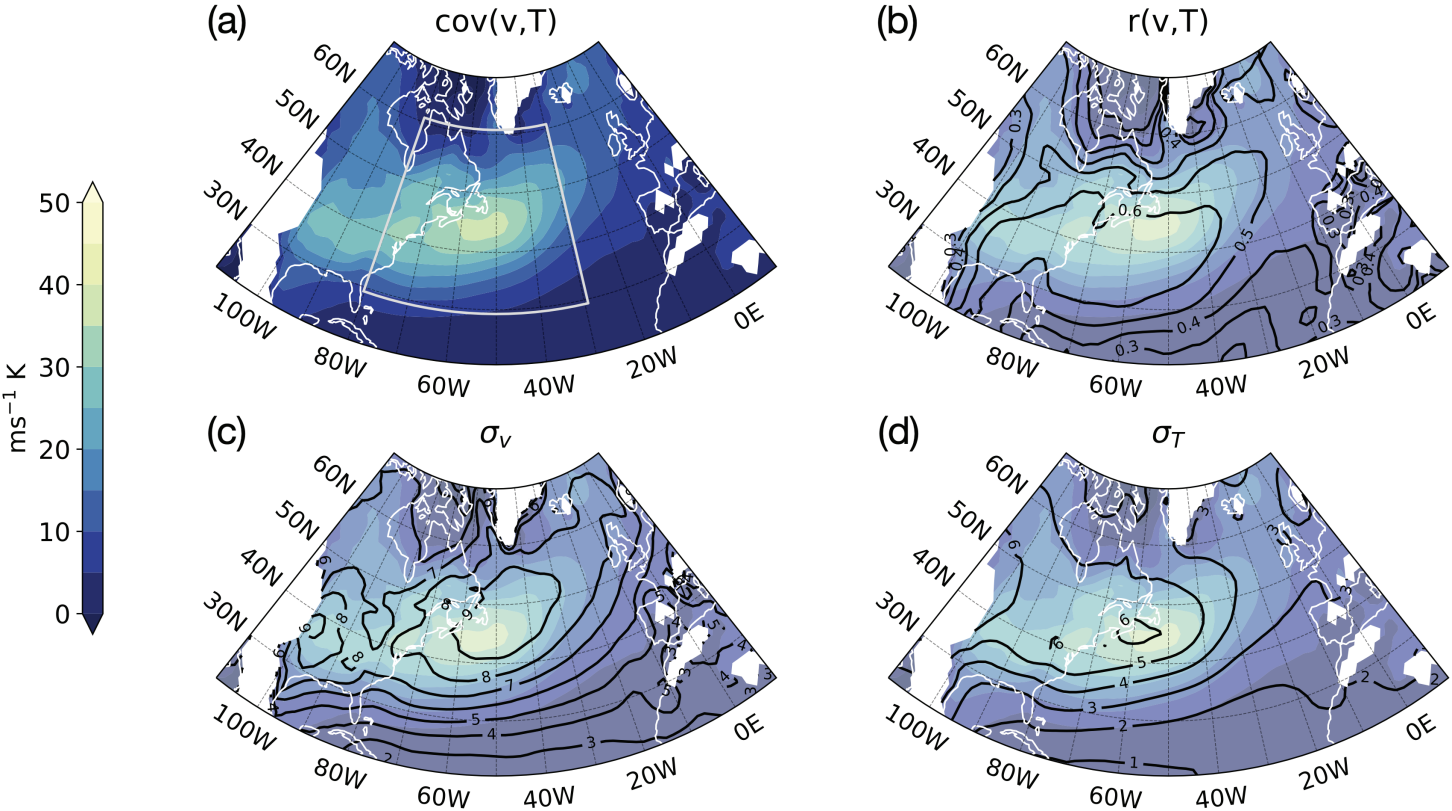
Decomposition of synoptic‐scale *v*–*T* time covariance (Equation 2) into its components. (a–d) show time covariance (colour shading), correlation and standard deviations of *v* and *T* (black contours) evaluated at the 850 hPa level, respectively. Colour shading in all panels represents *v*–*T* covariance (shading in (b–d) is faded to highlight solid contours). The area within the light grey contour in (a) corresponds with the spatial domain where $v^{\prime }$–$T^{\prime }$ spatial covariance is calculated (see text). Regions where orography is greater than 1000 m (white shading) are not included in our analyses [Colour figure can be viewed at wileyonlinelibrary.com]

Figure 1 shows the different components of synoptic‐scale *v*–*T* time covariance over the North Atlantic basin in winter, when storm activity is the most intense. Covariance is observed to peak along the major storm track region, which is consistent with the definition of storm tracks from a Eulerian point of view (Blackmon *et al*., 1977). What is more, we also notice that the spatial patterns for covariance and its components resemble each other, all reaching the highest values along the North Atlantic storm track, with the maximum in correlation slightly to the south of the maximum in covariance. A simple visual comparison between the spatial patterns of the components of covariance thus seems to suggest that stronger covariance is the result not only of larger variance but also of enhanced correlation.

**FIGURE 2:**
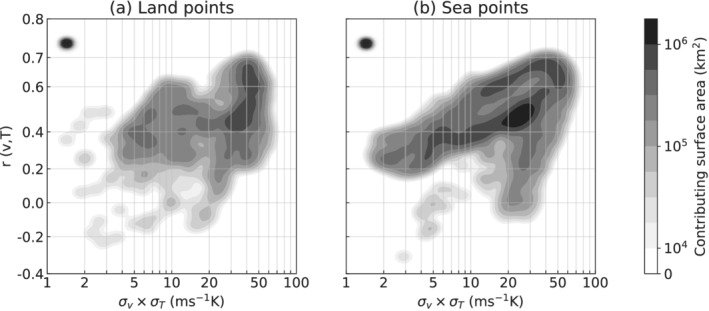
Kernel density estimate of the distribution of *v*–*T* time correlation against the product of $\sigma_{v}$ and $\sigma_{T}$ for (a) land‐covered and (b) sea‐covered grid points. Shading represents surface area extent contributing to each point. The black‐shaded dot at the left of the plots indicates the shape in the correlation–standard deviation space of each contributing point from Figure 1

This geographical correspondence is made even more evident when correlation *r* is plotted pointwise against the product of standard deviations $\sigma_{v}\sigma_{T}$, using values from Figure 1. In order to account for the different weight each point contributes in building the empirical density distribution, we perform a kernel density estimation (i.e., each point is assigned a Gaussian distribution function and then summed over all points; Appendix A gives the technical details) and multiply each contribution by the areal extent associated with it, which varies depending on its latitude. The resulting picture indicates the total area that contributes to each point in the correlation–variance space. The resulting distribution is shown in Figure 2, where we make a distinction between land and sea points. We notice that increased correlation systematically matches increased variance over sea surfaces (Figure 2b), while over land the relation is not as clear (Figure 2a). The sea points further display a secondary data cluster where high variances correspond to a range of correlation values.

From a statistical point of view, correlation between two variables is not expected to necessarily vary jointly with the standard deviation (or, equally, variance $\sigma^{2}$) of either of the variables. In fact, a simple addition of uncorrelated variance would lead to a reduction in correlation. Furthermore, if we consider a simple diffusive model for the relationship between meridional wind and temperature anomalies (i.e., $T^{\prime }\approx -\tau v^{\prime }\partial \hat{T}/\partial y$, where τ is a decorrelation time and $\hat{T}$ is the 10‐day running mean), we actually find that the resulting wind–temperature correlation increases significantly and independently of variances (not shown). It thus appears that the observed increase in correlation with variances is driven by some physical mechanism.

This hypothesis is also supported by the different behaviour over land and sea areas. The presence of a secondary data cluster in Figure 2b further suggests that the relationship between correlation and variance may be linked to the distinct dynamical characteristics of the atmosphere over different regions of the oceanic basins. Indeed, grid points contributing to the secondary data cluster of high variance values were found to be located predominantly in the polar regions of the North Atlantic basin, between Canada and Greenland (not shown).

Marcheggiani and Ambaum (2020) observed a similar behaviour in correlation and variances between surface heat flux and air temperature, suggesting that the simultaneous growth of correlation and variance could be a generic property of air–sea thermal interactions. Enhanced convection at the surface in cold sectors of extratropical weather systems leads to deeper atmospheric boundary layers, thus strengthening the coupling of the surface to the free troposphere. Nonetheless, it is not obvious how a related mechanism could be responsible for the increase in correlation between *v* and *T* and their variances.

## 3 PHASE SPACE ANALYSIS OF SPATIAL CORRELATION AND VARIANCES

The climatological mean picture considered above hides details of the dynamical evolution of the covariance between *v* and *T* on synoptic time‐scales. Therefore, we next take into consideration spatial variability and its evolution in time, which enables us to investigate the temporal evolution of covariance. Following Marcheggiani and Ambaum (2020), we construct a mixed space–time framework where we consider the spatial variances and correlation between time‐anomalous fields of *v* and *T* over a fixed spatial domain. Time anomalies of *v* and *T* are again defined as deviations from a 10‐day running mean and the spatial domain we selected broadly coincides with the Gulf Stream extension region ($30^{\circ }-60^{\circ }$N; $30^{\circ }-79.5^{\circ }$W) and is shown in Figure 1a. In our analyses, only non‐land grid points are taken into account in order to concentrate on the role the ocean plays in the dynamical evolution of correlation and variances, as Figure 2 suggested that the increase of correlation with variance is observed predominantly over sea surfaces.

**FIGURE 3:**
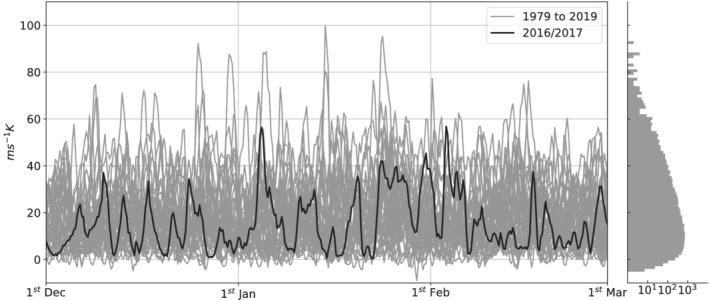
Time series of $v^{\prime }$–$T^{\prime }$ spatial covariance computed over the upstream region of the North Atlantic storm track (30–60°N, 30–79.5°W), spanning winters from 1979 to 2019 (grey solid lines) and highlighting a sample season (2016/2017, solid black line). To the right, the corresponding empirical distribution of the values of covariance is shown (semi‐log scale)

In this framework, the time‐evolving covariance, correlation and variances are related to each other analogously to Equation 1. Specifically, covariance is computed as the spatial average of the pointwise product of space–time anomalies in *v* and *T*,

(3) $$\begin{array}{ll} \text{cov}(v^{\prime },T^{\prime })=\langle v^{\prime *}T^{\prime *}\rangle & =\langle (v^{\prime }-\langle v^{\prime }\rangle )(T^{\prime }-\langle T^{\prime }\rangle )\rangle \\ & =\langle v^{\prime }T^{\prime }\rangle -\langle v^{\prime }\rangle \langle T^{\prime }\rangle , \end{array}$$

where primes denote time anomalies, angle brackets indicate the spatial average operator and asterisks deviations from this spatial average. We thus obtain a time series for the spatial covariance between *v* and *T* time anomalies for all winters from 1979 to 2019, which is shown in Figure 3. As expected, the temporal evolution of the $v^{\prime }$–$T^{\prime }$ covariance is characterised by intermittent bursts of activity (or peaks) that alternate with periods of weaker amplitude variability, which is reflected in the corresponding empirical distribution shown on the right of Figure 3 (mode below 10 m·s${}^{-1}$K, extensive tail towards higher values).

To corroborate our interpretation of $v^{\prime }$–$T^{\prime }$ covariance as a measure of meridional heat flux, we construct a phase space where the coordinate axes correspond to $v^{\prime }$–$T^{\prime }$ covariance and local mean baroclinicity. Therein, we plot the time series for covariance against that for the mean lower‐tropospheric baroclinicity, measured as the maximum Eady growth rate at 750 hPa (Hoskins and Valdes, 1990) and spatially averaged across the region marked in Figure 7 below. We then apply a Gaussian kernel smoother in the phase space and obtain a phase portrait for their average co‐evolution. The size of the Gaussian kernel can be adjusted to filter out small‐scale features due to the intrinsically chaotic nature of the systems and evince the main circulation in the phase space.

Kernel filtering is employed exclusively in the phase space and no time filtering is applied to the raw data series used to build phase portraits, save the removal of a 10‐day running mean in the computation of the time anomalies in *v* and *T*. There are several examples in recent literature (e.g., Novak *et al*., 2017; Marcheggiani and Ambaum, 2020; Yano *et al*., 2020) for the use of kernel averaging and phase space analysis to examine the dynamical evolution of chaotic nonlinear weather and climate systems. Novak *et al*. (2017) provides a thorough description of kernel averaging in a phase space that we employ in this study (in particular, their Figure 4; also Appendix A here).

**FIGURE 4:**
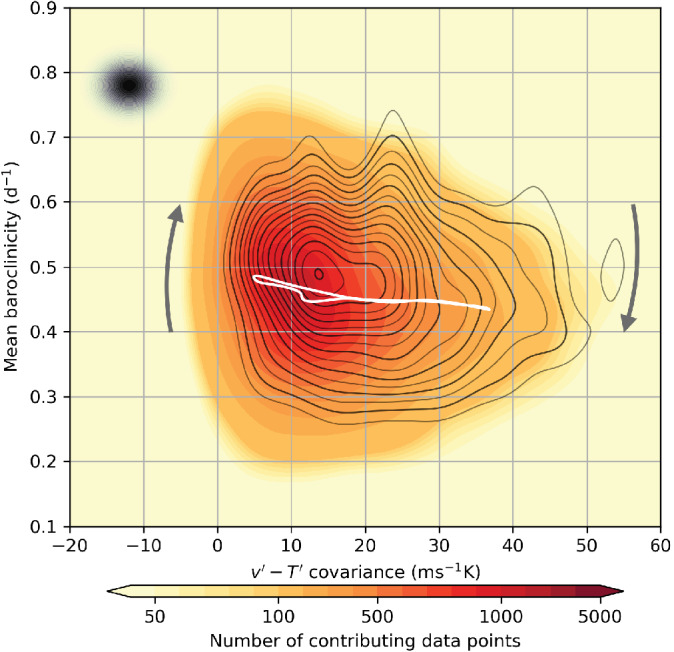
Kernel‐averaged circulation in the $v^{\prime }$–$T^{\prime }$ spatial covariance–baroclinicity phase space. Contours represent lines of constant streamfunction and the arrows the direction of the flow. The size of the averaging Gaussian filter is indicated by the black‐shaded dot at the left of the plot. The white line represents the projection onto this phase space of the kernel‐averaged baroclinicity along the closed trajectory marked in Figure 6 (Section 4 gives details) [Colour figure can be viewed at wileyonlinelibrary.com]

The picture of the average circulation in the phase space that we obtain, shown in Figure 4, is very similar to the phase portrait of heat‐flux–baroclinicity presented by Novak *et al*. (2017) in their Figure 5. It is also consistent with the predator–prey relationship highlighted in the same study, whereby meridional heat fluxes *feed* on mean background baroclinicity, which can only recover when heat fluxes are weak.

We thus find a clockwise mean circulation in phase space where in quiescent periods the baroclinicity builds up to exceed a critical value (about $0.5\;{\text{day}}^{-1}$) after which the $v^{\prime }$–$T^{\prime }$ covariance shoots up and at the same time starts to erode the baroclinicity because of the attendant reduction in temperature gradient due to the downgradient heat fluxes. When the baroclinicity has reduced below criticality, the $v^{\prime }$–$T^{\prime }$ covariance starts to decay and the cycle starts again.

The $v^{\prime }$–$T^{\prime }$ covariance is seen to be positive most of the time, with a small fraction of events associated with negative correlation. This is only partly an artefact of kernel averaging, as the raw data also show occasional negative correlations for short periods of time.

**FIGURE 5:**
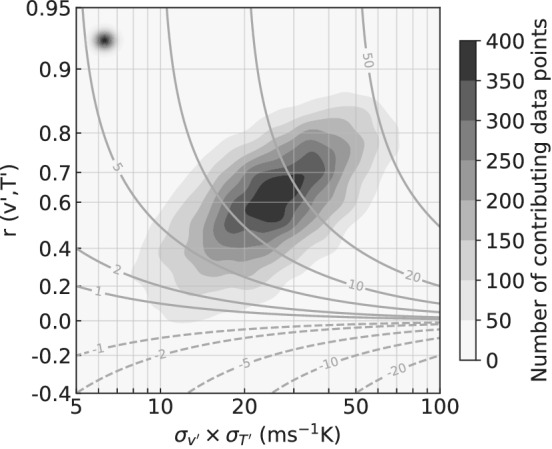
Kernel density estimate of the distribution of $v^{\prime }$–$T^{\prime }$ spatial correlation against the product of $\sigma_{v^{\prime }}$ and $\sigma_{T^{\prime }}$. Contours represent $v^{\prime }$–$T^{\prime }$ spatial covariance and shading the number of points contributing to the kernel average. The size of the averaging Gaussian filter is indicated by the black‐shaded dot at the left of the plot

Similar to what was observed from the time‐only perspective presented in Section 2, we find that for an increase of $v^{\prime }$–$T^{\prime }$ covariance, the spatial correlation and variances are seen to increase at the same time. In Figure 5, we plot spatial correlation against the product of standard deviations in $v^{\prime }$ and $T^{\prime }$. Despite the large spread in the data distribution, Figure 5 suggests that higher values of spatial correlation occur more frequently at higher variances, while lower variance is typically associated with weaker correlation. This provides further evidence of the existence of a physical process that ties the change in variance to the change in correlation.

## 4 THE LIFE CYCLE OF $v^{\prime }$–$T^{\prime }$ COVARIANCE

Further understanding of the dynamical relationship between correlation and variances can be achieved through the construction of a correlation–variances phase space, thus investigating the evolution of covariance in terms of its components.

The calculation of $v^{\prime }$–$T^{\prime }$ spatial covariance follows from Equation 3 and consists essentially of the sum of the products of *v* and *T* departures from the area‐mean values over all the grid‐points within the chosen spatial domain. The sum is weighted according to the area represented by each grid point, which is proportional to the cosine of its latitude. We choose to represent the fields for meridional wind and temperature time anomalies in the form of vectors whose components correspond to every grid point in the longitude–latitude grid and we can thus write the spatial covariance between $v^{\prime }$ and $T^{\prime }$ at any time *t* as the weighted inner product between these two vectors,

$$\begin{array}{llll} \text{cov}(v^{\prime },T^{\prime }){|}_{t} & =\overset{N}{\underset{i}{\sum }}\;v_{i}^{\prime }\;T_{i}^{\prime }\;{\tilde{w}}_{i}\; & & \\ & ={\text{v}}^{\prime }\cdot {\text{T}}^{\prime }=\|{\text{v}}^{\prime }\|\;\|{\text{T}}^{\prime }\|\cos\phi ,\; & & \end{array}$$

where *N* is the total number of grid‐points making up the spatial domain considered, ${\tilde{w}}_{i}=w_{i}/\sum_{i}^{N}w_{i}$ are the normalised weights proportional to the area each grid‐point represents, and ϕ is the angle between the vectors (characters in bold). The weighted inner product between two vectors is defined as the dot product of the two vectors after a pointwise multiplication with the weights vector $\text{w}=(w_{1},w_{2},...)$. The weighted inner product then induces a norm $\|{\text{v}}^{\prime }\|=\sqrt{{\text{v}}^{\prime }\cdot {\text{v}}^{\prime }}$ which we can interpret as the spatial standard deviation of $v^{\prime }$ ($\sigma_{v^{\prime }}$) at time *t* and, analogously, $\|{\text{T}}^{\prime }\|=\sigma_{T^{\prime }}$. The angle ϕ that vectors ${\text{v}}^{\prime }$ and ${\text{T}}^{\prime }$ form between each other is related to the spatial correlation between $v^{\prime }$ and $T^{\prime }$,

(4) $$r(v^{\prime },T^{\prime }){|}_{t}=\frac{{\text{v}}^{\prime }\cdot {\text{T}}^{\prime }}{\left\|{\text{v}}^{\prime }\|\;\|{\text{T}}^{\prime }\right\|}=\cos\phi .$$

The vectors ${\text{v}}^{\prime }$ and ${\text{T}}^{\prime }$ share the same dimensionality (i.e., number of grid‐points considered, in our case) which guarantees that ϕ is an angle.

This representation of covariance then suggests a way to plot the different components of covariance in a two‐dimensional space in polar coordinates, with the radial and azimuthal coordinates corresponding to $\left|{\text{v}}^{\prime }|\;|{\text{T}}^{\prime }\right|$ and $\phi =\cos^{-1}r$ respectively. In this space, covariance increases linearly in the horizontal (*x*) direction, being the product of the radial coordinate and the cosine of the azimuthal coordinate. The space itself is isotropic with the *x* and *y* directions having the same physical dimension ($\left|{\text{v}}^{\prime }|\;|{\text{T}}^{\prime }\right|$). By the same token, the distance between two points in this space would be given by their Euclidean distance rather than the difference in their $v^{\prime }$–$T^{\prime }$ covariance.

**FIGURE 6:**
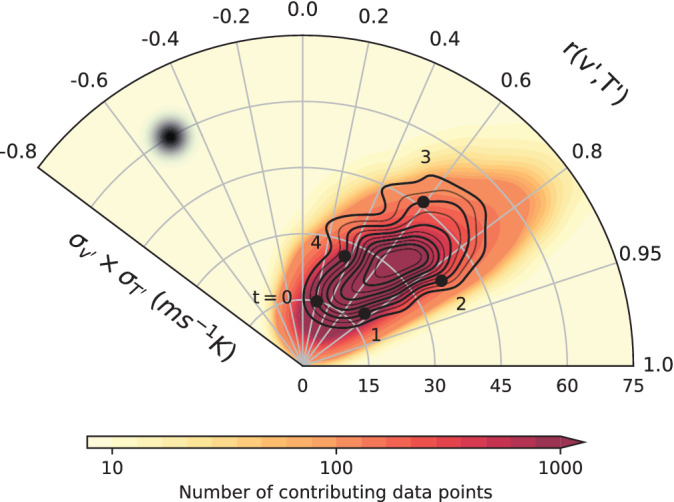
Kernel‐averaged circulation in the spatial variance–correlation phase space (based on the same data series as in Figure 5, using a polar coordinate system). The radial coordinate corresponds to the product of $\sigma_{v^{\prime }}$ and $\sigma_{T^{\prime }}$, the cosine of the azimuthal coordinate to $r(v^{\prime },T^{\prime })$ according to Equation 4. Contours represent lines of constant streamfunction, defined by Equation 5. The flow in the phase space is in the anticlockwise direction. See text for labels along one of the contour lines. The size of the averaging Gaussian filter is indicated by the black‐shaded dot at the left of the plot [Colour figure can be viewed at wileyonlinelibrary.com]

Time series for $\sigma_{v^{\prime }}\times \sigma_{T^{\prime }}$ and $r(v^{\prime },T^{\prime })$ are plotted against each other and the resulting picture smoothed by taking a Gaussian kernel average to filter out small‐scale noise. A streamfunction ψ of the resulting circulation can be defined such that:

(5) $$u_{r}=\frac{1}{r}\frac{\partial \psi }{\partial \phi },\;u_{\phi }=-\frac{\partial \psi }{\partial r},$$

where $u_{r}$, $u_{\phi }$ denote the radial and azimuthal phase speeds respectively. The visualisation of the stream function helps evince the correlation–variance dynamical co‐evolution, as can be seen in Figure 6, which is the polar‐coordinate version of Figure 5. There, contours of ψ are plotted along with the number of data points (in shading) contributing to the kernel average at each point in the phase space. (Appendix B gives a discussion of the statistical significance of the kernel‐averaged circulation in Figure 6 .)

Data are almost entirely distributed in the positive‐correlation sector of the phase space and the few negative instances are partly an artefact of kernel averaging. Furthermore, the average circulation is in the anticlockwise direction, with increases in $v^{\prime }$–$T^{\prime }$ covariance occurring on average at high correlations (around 0.9), while decreases in covariance occur at lower correlation values (around 0.5). This suggests that a higher level of correlation is crucial to the build‐up of variance, and covariance more in general.

To further our understanding of the mechanisms at play in the evolution of $v^{\prime }$–$T^{\prime }$ covariance, we explore the dynamics associated with the circulation in the phase space. To this effect, we identify a closed trajectory in the kernel‐averaged circulation by selecting a stream function isoline, ensuring it crosses regions of high data density in order to retain robustness in the analysis results. We take as reference starting point of the chosen trajectory, namely day 0, the minimum in $v^{\prime }$–$T^{\prime }$ covariance. It takes about 5 days (4.7 days) for a complete revolution along this trajectory, which is outlined in Figure 6, where each day is marked with a black dot. Along this trajectory, one may distinguish four separate stages in the evolution of covariance:

1. Covariance build up with increasing correlation at low variances (days 0–1);
2. Increasing variance at high correlation (days 1–2);
3. Peak covariance as variances keep increasing while correlation starts to decay (days 2–3);
4. Covariance decay with both decreasing variances and correlation (days 3–0).

While demarcation points between different stages are somewhat subjective, the results of our analysis are not susceptible to minor changes in the above partition. We should point out that the time reference we take does not map trivially onto the evolution of a single system as it corresponds to the drift speed in the correlation–variances phase space. It takes on average five days for a complete cycle, however individual stages might last longer or shorter in actual events.

At each point along the trajectory we calculate the kernel average of geopotential height at 1,000 hPa (Z1000) and 500 hPa (Z500). The choice of Z1000 and Z500 fields is meant to help visualise the structure of the atmospheric flow at multiple levels while also bearing information about temperature advection occurring between the two levels, as temperature advection between Z1000 and Z500 is proportional to the Jacobian of Z1000 and Z500.

**FIGURE 7:**
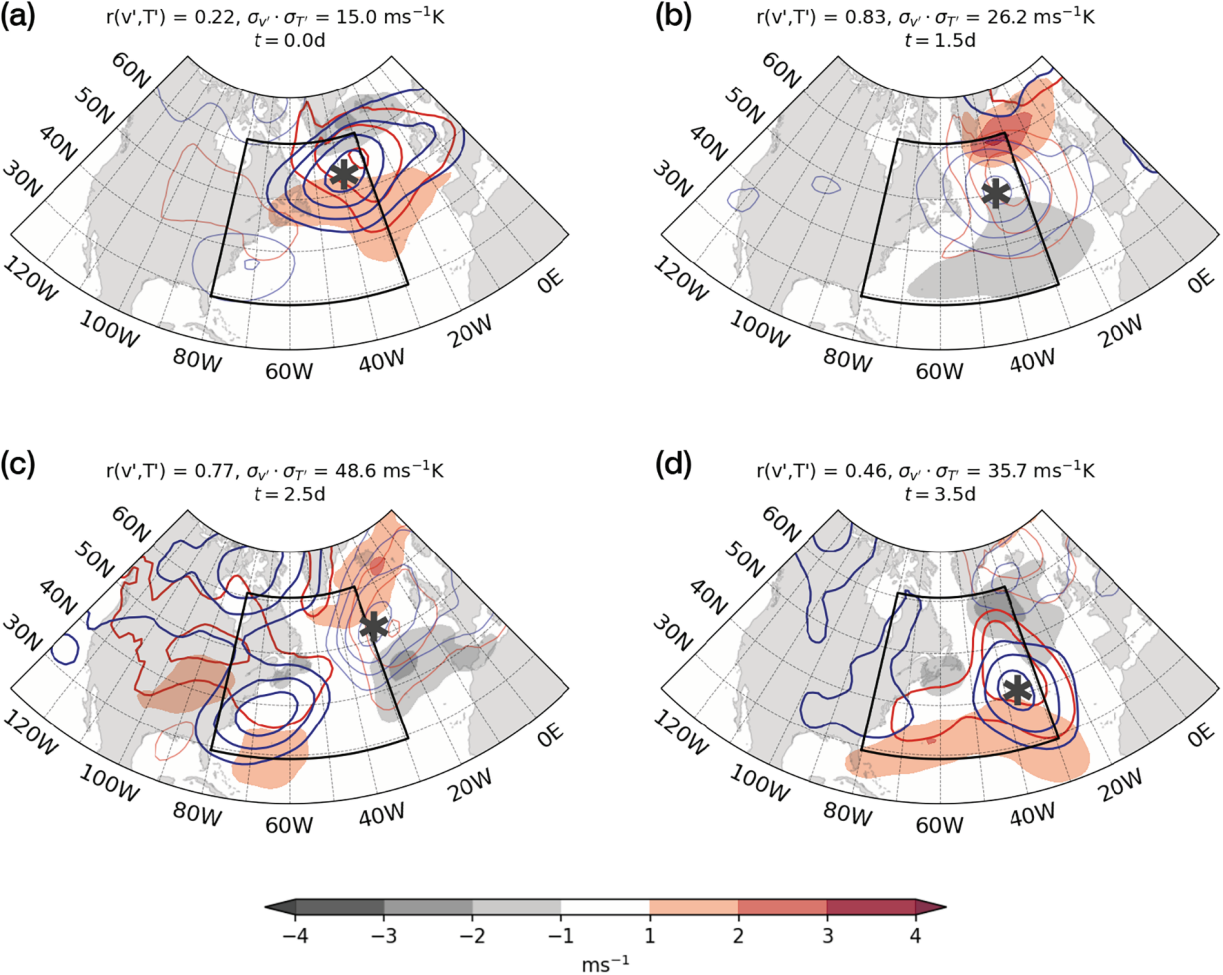
Kernel‐averaged composites of Z1000, Z500 and zonal wind (vertically averaged between 950 and 750 hPa) minus climatology for days (a) 0, (b) 1.5, (c) 2.5 and (d) 3.5. Contours of Z1000 and Z500 (red and blue, respectively, in the colour version) are plotted every 1 m, omitting 0 m contours; negative contours are plotted in bold. Shading represents zonal wind anomalies. Stars indicate crests of a propagating Rossby wave (see text for details) [Colour figure can be viewed at wileyonlinelibrary.com]

The resulting kernel‐averaged picture of the circulation in the phase space is shown in Figure 7. Although the choice of a specific closed trajectory is somewhat arbitrary, our qualitative results are not sensitive to this choice and the use of different closed trajectories resembling the one in Figure 6 leads to a similar evolution as that portrayed in Figure 7 (not shown). Each composite in Figure 7 represents the average of a large number of events, whose contribution is weighted according to the difference between their associated correlation and variances and those of the point where the kernel average is being computed. Systems with similar correlation and variances do not necessarily coincide geographically but contribute equally to the average, so that specific, smaller‐scale features would typically be averaged out and the resulting picture highlights the typical large‐scale structure of the flow at each stage in the evolution of a meridional heat flux peak. Therefore, although the composites are ineluctably affected by some degree of noise, it is nonetheless possible to relate the large‐scale picture to the life‐cycle viewpoint discussed above.

In the first stage of the life cycle, the flow is initially mostly aligned with the zonal direction within the spatial domain considered, while it veers to the north further downstream (Figure 7a). We observe that the average vertical structure of systems feature a south to southwest tilt (see centres of negative anomalies to the southeast of Greenland). The vertical tilt in geopotential is not conducive to poleward heat flux even in the presence of a westward tilt as it also features a marked southward component. The largest, negative anomalies in geopotential height are located in the northeastern corner of the spatial domain and further downstream in the eastern North Atlantic, which points to the predominance of synoptic variability (associated with the amplitude of a propagating Rossby wave, as we discuss further below) downstream of the storm track during this stage. We also notice a weaker high‐pressure anomaly in the southwestern sector of the domain, which will intensify as it propagates northeastward in the transition to the second stage.

In the transition from first to second stage, after correlation has reached larger values (above 0.5), variances slowly increase. The increase of variance at high correlation is indicative of baroclinic growth of synoptic eddies, as synoptic disturbances typically develop and evolve along the region of enhanced low‐level baroclinicity that is co‐located with the strong SST gradients associated with the Gulf Stream. Given the large spatial extent of the study region, the spatial distribution of the occurrence of synoptic systems is sporadic and the correct representation of their intensity may be hidden by the composition of a kernel‐averaged picture. However, the distinctive baroclinic structure is captured in the region in the later part of the first life‐cycle stage (not shown) and emerges more evidently in the transition to the next stage, as the vertical tilt in geopotential becomes more aligned in the east–west direction.

In the second life‐cycle stage, the rise in the variances' magnitude becomes the predominant mechanism in driving the increase in $v^{\prime }$–$T^{\prime }$ covariance (Figure 6, days 1–2). The low‐pressure system that was dominant in the first stage leaves the spatial domain, where anomalous high pressure now dominates (Figure 7b), possibly having evolved from the weaker positive anomaly off the eastern coast of the North American continent seen in Figure 7a.

The third stage corresponds with covariance reaching its peak value (43–44 m·s${}^{-1}$K), while correlation starts decaying after t=2.0 days, having attained the highest values between t=1.5 and 2.0 days. Around t=2.5 days (Figure 7c), the baroclinic structure of the flow is also evident, as the vertical tilt in geopotential at this stage is aligned mostly west to east. At the same time, the strongest temperature advection is seen to occur, which is consistent with the peak in meridional heat transport as measured by the $v^{\prime }$–$T^{\prime }$ spatial covariance.

In the fourth and final stage, $v^{\prime }$–$T^{\prime }$ covariance decay is primarily associated with the variances decreasing at low correlation. The flow in this stage (Figure 7d) is characterised by the decay of the previously noted baroclinic system, as it transitions back to the onset state of minimum correlation and variances (Figure 7a).

The later life‐cycle stages (third and fourth) are less well‐defined, as different physical mechanisms, often associated with nonlinear wave‐breaking, might be driving the breakdown of correlation and variances. The kernel‐averaged composites in Figure 7 are shaped by the contribution from these different mechanisms, which arguably makes it difficult to follow the average evolution of the flow.

The development and subsequent evolution of geopotential anomalies across the four stages of the covariance life cycle can be understood in terms of Rossby wave propagation. Rossby wave propagation dominates in the midlatitudes and our study suggests that peaks of meridional heat flux are most clearly linked to Rossby wave propagation than to Lagrangian propagation of individual weather systems, which was found to be dominant in the evolution of surface heat‐flux–temperature covariance (Marcheggiani and Ambaum, 2020). In fact, it is difficult to identify and track the evolution of specific features in the composites of the atmospheric flow at different points in the life cycle of $v^{\prime }$–$T^{\prime }$ covariance, while the transition from one stage to the other is reminiscent of stationary Rossby waves propagating along the North Atlantic waveguide described in Hoskins and Ambrizzi (1993). In particular, we would not expect large‐scale Rossby waves to be averaged out across different stages in the phase‐space evolution, because these large‐scale waves are quasi‐stationary and make up the planetary wave structure.

An example of Rossby wave propagation can be seen in the transition from the third stage to the fourth (Figure 7c,d): the peak intensity of the Rossby wave packet is seen to propagate downstream due to the eastward group speed while the phase speed appears to be mostly stationary as the corresponding centres between panels do not move much in the longitudinal direction (compare centres of high and low geopotential anomalies between 25 and 35°W, indicated respectively by stars in panels c and d). The change in sign of the anomalies with largest amplitudes is associated with group propagation rather than phase propagation as crests and troughs of higher wave numbers remain fixed in space and change sign depending on the propagation of the envelope of the wave packet.

Another example of this propagation mechanism can be found in the second stage of the life cycle, where the development of the large positive geopotential height anomaly in Figure 7b can also be interpreted as the result of Rossby wave propagation of the low‐amplitude positive anomaly taking shape in the southwestern sector of the spatial domain in Figure 7a. From examining composites at various intermediate stages (not shown), we can confirm that the evolution of the composites in the phase space is consistent with this Rossby wave propagation mechanism, rather than advection of a weather system: the individual centres do not move much (stars in Figure 7a, b) but the wave activity propagates downstream.

We also find that in the initial, growing stage the Rossby wave propagation is more along the southwest–northeast axis, while in the decaying phase the propagation is more along the west–east axis. This appears consistent with a general northward tilting of the waveguide during the heat flux events perhaps following the general northward tilting of the low‐level jet stream (Franzke *et al*., 2011; Novak *et al.,* 2015). Hoskins and Ambrizzi (1993) also show that especially over the North Atlantic region the Rossby wave propagation can be quite dispersed with distinct centres of action both in the northeast and the southeast directions.

The different configurations of the flow following the evolution of $v^{\prime }$–$T^{\prime }$ covariance are reminiscent of the three most persistent regimes of the North Atlantic eddy‐driven jet, namely the southern, central and northern jet states as identified by the jet latitudinal position (Woollings *et al*., 2010). Each regime is associated with distinct stages in the evolution of the storm track and in the dominant type of Rossby wave breaking, mostly cyclonic and anticyclonic in the southern and northern regimes, respectively, while the central regime is influenced by both (Novak *et al*., 2015). In particular, Franzke *et al*. (2011) showed that the preferred transitions across the different regimes are from southern to central, from northern to southern and from central to northern, which is suggestive of an average poleward propagation of the eddy‐driven jet in the cyclical evolution of the jet.

Novak *et al*. (2015) linked the transitions across the three jet regimes to the different stages in the life cycle of the North Atlantic storm track by drawing a parallel with the predator–prey cyclical relationship between heat fluxes and baroclinicity, as predicted by the nonlinear model proposed in Ambaum and Novak (2014). Messori *et al*. (2017) in turn linked this nonlinear relationship to the temporal variability of the meridional heat transport.

We can draw an analogy between the evolution of the system in the correlation–variances phase space and that of the eddy‐driven jet latitudinal variability by concentrating on the structure of the anomalous flow portrayed by the kernel composites in Figure 7.

- Initially (Figure 7a), the picture that results from kernel averaging is comparable to that associated with the central regime of the jet's latitudinal position (Figure 4 in Woollings *et al.,* 2010), which gradually shifts northwards over the following day in the life cycle.
- As we enter the second stage of the life cycle, we can observe the transition from the central to the northern regime, as high pressure becomes predominant, the flow is deflected northwards and the maximum zonal wind moves northwards to around 60°N (Figure 7b).
- During the third stage, $v^{\prime }$–$T^{\prime }$ covariance and meridional temperature advection is largest (Figure 7c) and negative anomalies in geopotential height start to build up, chiefly in the southwest quadrant of the North Atlantic, which reflects the abrupt transition from the northern to the southern regime in the jet latitudinal variability, as positive zonal wind anomalies in the southern sector of the domain start to appear and intensify in the later part of the third stage (Figure 7d).
- Finally, the jet gradually moves back to the initial central regime during the course of the fourth stage we identified (Figures 7d,a), which is arguably the least well‐defined, as several different dynamical processes might be simultaneously at play (e.g., the influence of the subtropical jet in the southern regime or non‐modal system growth).

The evolution of the flow during the life cycle of $v^{\prime }$–$T^{\prime }$ covariance is thus consistent with the preferred regime transitions (i.e., southern $\overset{}{\underset{}{\to }}$ central $\overset{}{\underset{}{\to }}$ northern $\overset{}{\underset{}{\to }}$ southern) observed for the eddy‐driven jet (Novak *et al.,* 2015).

**FIGURE 8:**
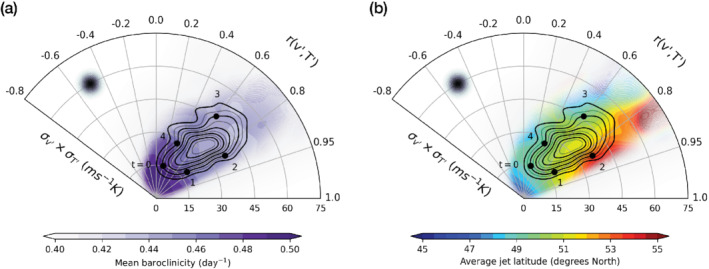
Kernel average (shading) of the (a) area‐mean baroclinicity and (b) eddy‐driven jet latitudinal position. Contours are as in Figure 6. The size of the averaging Gaussian filter is indicated by the black‐shaded dot in the upper‐left corner of both panels [Colour figure can be viewed at wileyonlinelibrary.com]

In Figure 8a we show the average evolution of the area‐mean baroclinicity (over the same spatial domain used to compute the covariance) across the correlation–variances phase space, while in Figure 8b we show that of the jet's latitudinal variability index. The jet latitude index is computed in a similar way to Novak *et al*. (2015), that is as the latitudinal position of the maximum in lower‐tropospheric (950 to 750 hPa) zonal wind, zonally averaged between $60^{\circ }$W and $0^{\circ }$E excluding regions covered by land. Baroclinicity is observed to decay on average during the build‐up of $v^{\prime }$–$T^{\prime }$ covariance, particularly as variances amplify, reaching a minimum at peak values in $v^{\prime }$–$T^{\prime }$ covariance. At the same time, the eddy‐driven jet is seen to gradually shift northwards in the first and second stages of the covariance life cycle, while it is located at lower latitudes in the decaying stage, consistent with the average evolution of the flow shown in Figure 7.

The observed variability in baroclinicity is limited compared to its full range of variability within the North Atlantic storm track seen in Figure 4, where in white we plotted covariance against the kernel‐averaged baroclinicity along the closed trajectory which we selected in the correlation–variances phase space (Figure 6). This might be indicative of the higher degree of complexity in the relationship between $v^{\prime }$–$T^{\prime }$ covariance and the storm track life cycle compared to what correlation–variances phase portraits convey, as the covariance life cycle does not map entirely on a baroclinic life cycle, especially in the final decay stage where different driving mechanisms might be at play. Furthermore, the level of noise associated with the kernel average is not negligible compared to the magnitude of the emerging signal in baroclinicity. However, we notice that baroclinicity values for the region considered are normally distributed around their mean (not shown), with a sample standard deviation (≈0.1 day${}^{-1}$) of comparable size with the amplitude of the variability observed in Figure 8a (ranging between 0.43 and 0.48 day${}^{-1}$). Thus, the resulting signal can be interpreted at least qualitatively as the average response of baroclinicity to $v^{\prime }$–$T^{\prime }$ covariance variability.

On a similar note, notwithstanding the fact that the full range of latitudinal variability spanned by the eddy‐driven jet is clearly larger than that associated with the $v^{\prime }$–$T^{\prime }$ covariance life cycle shown in Figure 8b, the clear signal of the jet's northward progression supports a physical link between jet latitude and the evolution of $v^{\prime }$–$T^{\prime }$ covariance over the Gulf Stream extension region. Finally, it should be noted that the difference in the time‐scales of amplitude variability in $v^{\prime }$–$T^{\prime }$ covariance (associated with high‐frequency eddy activity) and the downstream jet meandering can also be contributing to the limited magnitude of the average signal.

## 5 CONCLUSIONS

In this study we examined the temporal evolution in of the spatial covariance between meridional wind speed and air temperature as a tool to understand the dynamics underlying local heat transport variability. Specifically, we took an approach similar to that introduced by Marcheggiani and Ambaum (2020) and considered the spatial covariance between synoptic‐scale (2–10 days) time anomalies in meridional wind speed and air temperature over the western sector of the North Atlantic ocean. We found that $v^{\prime }$–$T^{\prime }$ spatial covariance (i.e., the spatial covariance between meridional wind and air temperature time anomalies) features frequent bursts of activity, reminiscent of the sporadic nature of meridional energy transport described in Messori and Czaja (2013), and its dynamical relationship with mean baroclinicity is consistent with recent studies on storm track variability (Novak *et al*., 2015; 2017).

We further noticed that $v^{\prime }$–$T^{\prime }$ correlation and variances increase jointly in the build‐up to strong covariance. This was also observed for covariance in time, especially over oceanic regions. Correlation between two variables is defined as the ratio of their covariance to the product of their standard deviations, and would not be expected to change with variances on purely statistical grounds. This points to the idea that some physical processes are driving this behaviour. Our analyses suggest that these can be partly ascribed to the dynamics of baroclinic development and eddy modal growth, which is characterised by a fixed correlation between anomalies in meridional wind and temperature and corresponds with phase‐locked Rossby waves. The roles played by baroclinic development and modal growth appear to be particularly relevant in the build‐up stages of the covariance's cyclical evolution, while different mechanisms are likely to be at play in the decay stage.

An initial small increase in covariance occurs at low variance due to correlation increasing from low values up to around 0.9. Subsequently, variances start to grow at high correlation. These two growing stages are associated with baroclinic development further downstream of the midlatitude eddy‐driven jet and eddy modal growth at constant, high correlation that leads to the peak in covariance. After the peak in covariance, correlation rapidly decays, while variances remain high and eventually also decay at low correlation. This brings the local velocity and temperature back to their initial low‐covariance state, which we took as the returning point of this intermittent life‐cycle as it coincides with the area of largest data density.

We uncovered a link between the evolution of $v^{\prime }$–$T^{\prime }$ covariance and Rossby wave propagation in the analysis of the average evolution of the flow, which suggests that localised peaks of meridional heat flux precede Rossby wave propagation along the Atlantic waveguide (Hoskins and Ambrizzi, 1993). The examination of refractive indices for Rossby waves in the phase space could shed more light on the role of $v^{\prime }$–$T^{\prime }$ covariance in storm track dynamics.

Furthermore, we evinced a correspondence between the life‐cycle‐like evolution of covariance and the different regimes of the eddy‐driven jet's latitudinal variability as described in Woollings *et al*. (2010). Moreover, the jet's regime transitions observed in our analyses match with the preferred transitions described in Franzke *et al*. (2011) and Novak *et al*. (2015). This correspondence points to the fact that spatial covariance and its components can be seen as dynamical variables carrying information about the evolution of weather systems.

Further insights into the importance of baroclinic development in shaping the correlation–variances co‐evolution could be gained through the study of simple models of baroclinic instability and their skill in reproducing the different stages in the $v^{\prime }$–$T^{\prime }$ covariance life cycle as observed in our phase space analysis.

## AUTHOR CONTRIBUTIONS

**Andrea Marcheggiani:** conceptualization; formal analysis; investigation; methodology; writing – original draft. **Maarten H. P. Ambaum:** conceptualization; investigation; methodology; writing – review and editing. **Gabriele Messori:** investigation; writing – review and editing.

## Figures and Tables

**FIGURE B1:**
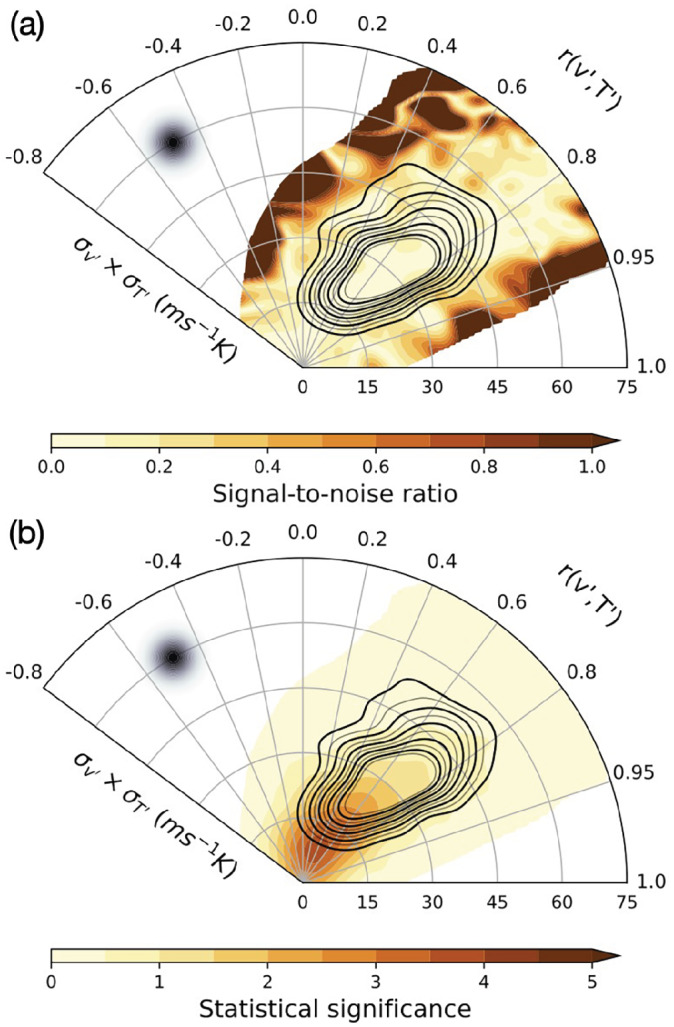
(a) Signal‐to‐noise ratio and (b) statistical significance computed as in Yano *et al.* (2020) for the phase velocities in the correlation–variances phase space, shown where the effective number of data points contributing to the average is greater than 1. Contours represent the streamfunction associated with kernel‐averaged circulation [Colour figure can be viewed at wileyonlinelibrary.com]

## Footnotes

1 We hereafter refer to the eddy transport of dry static energy as meridional heat flux.

## APPENDIX A KERNEL SMOOTHING

### A.1

Trajectories in phase portraits of real systems rarely follow the same path twice and often cluster around selected points or successions of points. On that account and given the large amount of data that are simultaneously visualised in this study (6‐hourly winter data from 1979 to 2019 amounts to more than 10,000 data entries), it becomes necessary to apply some level of filtering to the resulting phase portrait in order to evince the average circulation in the phase space.

Part of the noise due to the chaotic nature of the system can be filtered out by applying a kernel averaging operator (or smoother) to the raw data. The kernel averaging operator that we use in this study is based on a kernel density estimator (Wand and Jones, 1994 gives a detailed description ) and computes the sum of the contributions from every data entry weighted according to their distance from the point where the operator is applied. Mathematically, the kernel density estimator returns the average number of contributing data entries to the average value at any specific point in the phase space. In one dimension, the number of contributing data points *N* at a point $x_{0}$ along the *x*‐axis is

(A1)
$$N(x_{0};h)=\overset{n}{\underset{i=1}{\sum }}K_{h}(x_{0}-X_{i}),$$

where *n* is the number of discrete points that form the time series $X_{i}$, $K_{h}$ is the kernel (which is chosen to be a unimodal function, symmetric about zero) and *h* is a positive number controlling the window width (or bandwidth) of the kernel. The kernel in Equation A1 is chosen to be a Gaussian kernel,

(A2)
$$K_{h}(x)=\exp\{-\frac{1}{2}{\left(\frac{x}{h}\right)}^{2}\},$$

where *x* is the Eulerian distance between two points. While the particular choice of kernel is somewhat arbitrary, the results of our analyses should not be overly sensitive to the specific kernel implemented nor to its window width. Figure 5 in Novak *et al*. (2017) nicely presents the effect that using different bandwidths has on the kernel‐averaged phase space circulation.

To calculate the average value of a variable, *q*, across the phase space, we compute its kernel average by multiplying the value attained by *q* at each point in time by its weight (that is, its distance from the point where the kernel average is computed) and then divide by the estimated density. We thus obtain the kernel‐averaged value of the variable at each point in the phase space,

(A3)
$${\bar{q}}_{0}^{k}=\frac{1}{N(x_{0};h)}\underset{i}{\sum }K_{h}(x_{i}-x_{0},y_{i}-y_{0})\;q_{i}.$$

Here, ${\bar{q}}_{0}^{k}$ represents the kernel average at the point $\left(x_{0},y_{0}\right)$ in the phase space of the variable *q* and the sum is over all points $\left(x_{i},y_{i}\right)$ where the variable takes the values $q_{i}$. The kernel $K_{\text{h}}(x,y)$ is the extension in two dimensions of the one‐dimensional Gaussian kernel in Equation A2,

$$K_{\text{h}}(x,y)=\exp\left[-\frac{1}{2}\left\{{\left(\frac{x}{h_{x}}\right)}^{2}+{\left(\frac{y}{h_{y}}\right)}^{2}\right\}\right].$$

The parameter $h=(h_{x},h_{y})$ is equal to the product of $\sigma_{x,y}$ (standard deviations of the X,Y time series). The dimensionless parameters $f_{x,y}$ represent the fraction of the standard deviation of the full time series (of correlation or variances product, for instance) used to define the kernel, effectively tuning the size and shape of the Gaussian distribution corresponding to the kernel K(x,y). In our analyses we assume $f_{x}=f_{y}=f$ for the sake of simplicity, which means the parameter h becomes $h=(f\sigma_{x},f\sigma_{y})$.

## APPENDIX B STATISTICAL SIGNIFICANCE OF KERNEL‐AVERAGED PHASE PORTRAITS

### B.1

Yano *et al*. (2020) provide measures for both the signal‐to‐noise ratio and the statistical significance of the kernel‐averaged phase velocities. The signal‐to‐noise ratio is defined (Equation 4.1 in Yano *et al*., 2020) as the ratio between the size of the signal to the underlying spread, while the statistical significance is defined (Equation 2.10 in Yano *et al*., 2020) as the square root of the ratio of number of data contributing to the average (i.e., data density) and the underlying variance, in other words describing how many standard deviations the signal rises above the estimated expectation value of the noise.

In Figure B1 we show the signal‐to‐noise ratio and statistical significance for the kernel‐averaged circulation in the correlation–standard deviations phase space. The signal‐to‐noise ratio is well below 1 across the regions with highest data density, which implies that fluctuations of individual trajectories around the kernel‐averaged circulation are large, as expected: the signal represents a mean drift in a noisy background. However the statistical significance is quite high, particularly in the data‐dense regions, as panel b indicates, indicating that the small signal still stands out as significantly different from zero. In addition, we argue that the coherent structure of the phase space drift is independent evidence for the statistical robustness of the signal.
